# Is it Possible to Change Prescribing Habits?

**DOI:** 10.1371/journal.pmed.0020328

**Published:** 2005-09-06

In the US more than 770,000 people are injured or die each year in hospitals from adverse drug events (ADEs), which can cost a hospital, depending on its size, about US$5.6 million every year, excluding ADE-associated costs for malpractice and litigation and the personal costs of injuries to patients. Nationally, hospital expenses to treat patients who have ADEs during hospital admission are enormous: between $1.6 billion and $5.6 billion annually. The cost to patients is also high and not just monetary: those who have an ADE spend on average 8–12 days longer in the hospital than patients who do not have an ADE, and their admission costs $16,000 to $24,000 more.

One way for hospitals to tackle the problem of medication errors is to install computerized monitoring systems, which can reduce ADEs by 28%–95%. Apart from the obvious benefits to patients, these systems can save hospitals as much as $500,000 annually in direct costs. However, despite the potential, fewer than 10% of hospitals have implemented such systems.

Less is known about the value of such systems in an outpatient setting. Now, Andrew Steele and colleagues from Denver have tested a computerized physician order entry (CPOE) system in a US hospital's outpatient clinic. The main purpose of the study was to determine the impact of using computerized alerts to improve the prescribing of medications in the outpatient setting. Studies have shown that 18%–25% of patients might have an ADE in the outpatient environment. This study evaluated a CPOE system alongside an integrated computer-based clinical-decision support system.

It focused on a very specific type of clinical-decision support system: the use of a rules technology to prevent drug–laboratory ADEs. The way the system worked was that providers ordered medications on a computer and an alert was displayed if a relevant drug–laboratory interaction existed.

Comparisons were made between baseline and post-intervention periods. Provider ordering behavior was monitored, focusing on the number of medication orders not completed and the number of rule-associated laboratory test orders initiated after alert display. The investigators found that the rule processed 16,291 times during the study period on all possible medication orders: 7,017 during the pre-intervention period (prescribing doctors did not receive alerts) and 9,274 during the post-intervention period (prescribing doctors received alerts). During the post-intervention period, an alert was displayed for 11.8% (1,093 out of 9,274) of the times the rule processed, with 5.6% of alerts being for “missing laboratory values,” 6.0% for “abnormal rule-associated laboratory” values, and 0.2% for both types of problems.

Providers did pay attention to the alerts; they increased ordering of the rule-associated laboratory test when an alert was displayed (39% at baseline versus 51% post-intervention, *p* < 0.001), thus showing that the rules had a significant ability to change the ordering behavior of the provider, said the authors. The strongest effect occurred when providers where alerted to “missing” laboratory results (42% increase), the investigators noted. There was less of an effect on ordering behavior when the alert informed the provider of the existence of an abnormal laboratory value (23% increase), which may imply that the cutoff values for the “abnormal” trigger were set too low, suggested the authors. However, there was only a modest effect on halting the ordering of medications, and this was limited to occasions in which the alert presented an abnormal laboratory value in which case there was almost a doubling in order cessation.

There are limitations to the study. For example, the intervention focused on a specific group of drug–laboratory interactions and thus the results may not be generalizable to other types of interventions. In addition, the setting was a single primary-care clinic outpatient setting within a large public-health-integrated health-care delivery system, and results may be different in other settings such as hospitals and private physician offices. However, changing prescriber practice at all is not easy to achieve and this approach thus warrants further research.

